# Bioterrorism: how prepared are we?

**DOI:** 10.3201/eid0504.990402

**Published:** 1999

**Authors:** D E Shalala

**Affiliations:** U.S. Secretary of Health and Human Services, USA.


### Bioterrorism: How Prepared Are We?

**Donna E. Shalala**
U.S. Secretary of Health and Human Services

Richard Preston's The Cobra Event, which he dedicates to public health professionals, weaves a chilling, but compelling tale about a lone terrorist's attack on Manhattan with a genetically engineered virus. Preston's thought-provoking novel raises a logical question: How do we successfully contain and combat the threat of bioterrorism? To meet this emerging threat, we must address four important challenges.

The first challenge is to be aware that an act of bioterrorism could happen. Its likelihood is entirely unknown, and an attack may never occur. However, we have seen terrorism emerge as one of the thorniest problems of the post-cold war era, and we have seen that terrorists are always searching for new weapons. We have already seen sarin nerve gas released in the Tokyo subway. Somewhere, sometime in the future, terrorists may well threaten to use, or attempt to use, a biological weapon against the United States. When discussing the possibility of a terrorist attack in the next few years, the president unequivocally stated, "This is not a cause for panic. It is cause for serious, deliberate, disciplined, long-term concern." In other words, we must not be afraid, but we must be aware.

Once we are fully aware that bioterrorism could happen, our second challenge is to be prepared. That is why the Department of Health and Human Services (HHS) is spending $158 million this fiscal year to prepare for bioterorrism and why the president has proposed increasing that investment by an additional $72 million in his Fiscal Year 2000 budget.

This investment will fund our ongoing Anti-Bioterrorism Initiative. To increase our level of preparedness, the initiative is expanding its activities in a number of key areas: surveillance, medical and public health response, building a stockpile of drugs and supplies, and research and development. We are improving and strengthening the U.S. public health surveillance network by enhancing our capability to detect and report outbreaks, conduct epidemiologic investigations, perform laboratory tests to identify biological agents, and communicate necessary information and advisories rapidly through electronic technology.

We are enhancing our medical and public health response capacity by spearheading an administrationwide effort to develop infrastructure at the local level by establishing in major American cities medical response teams to deal with the consequences of bioterrorism. We are also expanding our capacity to provide prophylaxis, medical care, and infection control on a massive scale. We are creating, and will be maintaining, an unprecedented national stockpile of drugs and vaccines for civilian use in case of a bioterrorist attack.

Finally, we are accelerating our research and development of rapid diagnostics, drugs, and vaccines, so we can more effectively address the threats and consequences of a bioterrorist attack. In addition, we will continue our work on the genome sequencing of organisms most likely to be used as bioweapons, so that we can not only quickly identify the biological agent, but also develop effective therapies. Our efforts in surveillance, medical and public health response, stockpile provision, and research and development will increase significantly our preparedness for bioterrorism.

If we want to be truly prepared, our third challenge is for the public health and medical communities to take the lead in our fight against bioterrorism. In a conventional terrorist attack, local "first responders," such as the police, firefighters, and paramedics, constitute the first line of defense. With bioterrorism, the public health and medical communities stand directly on the front lines. How well we respond to a threat or attack will depend on the preparedness of our public health and medical communities. For example, if a bioterrorist threat is issued—perhaps someone claims to have released a deadly pathogen in a public place—physicians must be able to recognize and report cases that come to attention in emergency rooms and doctors' offices; public health officials must be able to conduct investigations to establish the likely site/time of exposure, the size and location of the exposed population, and the prospects for secondary transmission; and appropriately traned laboratory personnel must be available to identify the biological agent. Whether the release of a bioweapon is announced or surreptitious, affected persons may not have symptoms for days or even weeks, and by then they would be geographically dispersed. Quarantine is not practical because only one biological agent—smallpox—is communicable. Even with smallpox, it would be impossible to know whom to quarantine because of the spread of disease by secondary transmission and the difficulty in accurately identifying those who have been exposed. A strong electronic communications network would be needed to piece together early reports, as well as epidemiologic and laboratory data, to determine what had happened so that public health and law enforcement officials can take prompt action. The Centers for Disease Control and Prevention would play an important role in this process because of its particular expertise in surveillance, infectious disease, and public health. Everyone—from the physicians who first see victims to the scientists who identify the infectious agents—must coordinate their efforts.

That brings me to the fourth, and final, challenge: We must all work together. In the fight against bioterrorism, the federal government, particularly HHS, has a leadership role. Among other things, we need to support state and local planning efforts, provide training at every level, develop an infrastructure for delivering mass medical care, and offer expertise to our communities.

This is a fight we certainly cannot win by ourselves. Across the board, we must forge new working partnerships among health, public safety, and intelligence agencies. We need unprecedented cooperation among the federal government, state and local health agencies, and the medical community. We must ensure that plans for managing the medical consequences of terrorist acts are well integrated and coordinated with other emergency response systems.

Close collaborative efforts are necessary also because microbes do not respect boundaries of culture, language, or territory. An act of bioterrorism cannot be contained by any national border or barrier. When it comes to microbes, we are not protected, in the words of the Indian poet Tagore, "by narrow domestic walls." Since these organisms recognize no boundaries, in our battle against them, neither can we. Because we share a common future, we must share a common resolve. As Dr. Gro Bruntland, the director-general of the World Health Organization, has said, when it comes to public health and safety, "Solutions, like the problems, have to be global..." As we work together to counter bioterrorism, we must pool our will and our resources to meet the challenges.

